# Adjustment in third culture kids: A systematic review of literature

**DOI:** 10.3389/fpsyg.2022.939044

**Published:** 2022-11-28

**Authors:** Emma Marchal Jones, Marnie Reed, Jens Gaab, Yoon Phaik Ooi

**Affiliations:** ^1^Division of Clinical Psychology and Psychotherapy, Faculty of Psychology, University of Basel, Basel, Switzerland; ^2^Department of Developmental Psychiatry, Institute of Mental Health, Singapore, Singapore

**Keywords:** TCK, child, adolescent, adjustment, systematic review, factors

## Abstract

Third Culture Kids (TCKs) are children of expatriates who live in a culture other than their country of nationality or their parent's country of nationality for a significant part of their childhood. Past research has indicated that adjustment is a key factor in the success of global mobility. However, current research in the area of TCK adjustment is lacking. This systematic review aims to present and summarize all available published scientific data on the adjustment of internationally mobile children and adolescents who relocate with their families. We aim to understand factors related to TCK adjustment, highlight lacking research areas, and define areas of interest for future research. The eligibility criteria for inclusion in the review were: traditional TCKs; aged 7–17 years; measures taken during the relocation; outcome variables of wellbeing, psychological adjustment or social adjustment, or socio-cultural adjustment or adjustment. An initial search across eight databases in December 2021 yielded 9,433 studies, which were included in COVIDENCE and reviewed independently by two researchers at each phase. We finally included 14 studies in this study, 10 of which presented quantitative data. Extracted quantitative and qualitative studies were abstracted, and the main findings are presented using a consistent grid of codes: an initial computerized lexical scan (Leximancer) of all included papers generated a preliminary list of topics and their frequencies. We refined these initial topics using the most prominent theories around the topics of TCK, adjustment, and the extracted theories from selected papers and created a codebook. Then we abstracted the quantitative data from the selected studies and organized the statistically significant findings according to the codes. Lastly, we abstracted and synthesized the findings from qualitative studies. Efforts were made to present the available data within a reading grid, which enhances the understanding of mechanisms specific to the sample population and also makes it apparent where more research is needed. Specifically, findings suggest a need for a more inclusive multi-trajectory adjustment model and a better definition of the ecological sample. The coding system for the extraction and analysis in this systematic review may be a guide for researchers planning future studies on TCK adjustment.

**Systematic review registration:**

https://www.crd.york.ac.uk/prospero/display_record.php?ID=CRD42020151071, identifier: CRD42020151071.

## Introduction

In 2021, there were an estimated 87 million expatriates worldwide (Finaccord, 2018). As approximately half of all expatriates relocate with a partner or child (Caligiuri and Bonache, 2016), understanding the challenges of global mobility for expatriates and their families is paramount to supporting this population.

Children of expatriates or Third Culture Kids (TCKs) are defined as children “accompanying one's parent(s) into a country that is different from at least one parent's passport country(ies) due to a parent's choice of work or advanced training” (Pollock et al., 2010, p. 44). TCK refers to the fact that these individuals grow up being influenced by three cultures: the heritage culture(s), the host-country culture(s), and the culture of expatriates and other TCKs. Although elements from each culture are assimilated into the TCK's life and identity, these individuals often have a greater sense of belonging with other TCKs and the international community rather than with the host or heritage culture (Pollock et al., 2010). TCKs, such as children of military, foreign service, corporate and missionary families, are distinctly different from other populations such as immigrants, refugees, and international adoptees (Pollock et al., 2010). Although these groups share the common experience of moving internationally, the transient nature of their stay and high-mobility patterns distinguish TCKs from other similar groups.

Extensive literature has highlighted the importance of positive adjustment during global mobility for expatriates and their families (e.g., Shaffer et al., 1999; Andreason, 2008; Takeuchi, 2010; Sterle et al., 2018). Expatriate adjustment is a complex process of change in various domains in response to a new environment and culture (Haslberger et al., 2014). Adjustment has been measured through constructs such as wellbeing, levels of satisfaction with self and the environment, psychological and emotional comfort, and the degree of fit and effectiveness between the person and their environment (Dawis and Lofquist, 1984; Taft, 1988; Black and Stephens, 1989; Haslberger and Brewster, 2009). While past adjustment theories (e.g., Berry, 1990, 1997; Searle and Ward, 1990) set the stage for research and provide a framework for understanding this concept, they do not encapsulate the full complexities of expatriate adjustment. The more recent 3-D Model of Adjustment (Haslberger et al., 2014) offers a more holistic view of adjustment by proposing an interplay between internal and external dimensions, several domains, and time. In the existing literature, expatriate adjustment is often measured in terms of psychological and socio-cultural adjustment. Psychological adjustment can be measured through indicators of wellbeing and mental health, such as internalizing (i.e., depression and anxiety) or externalizing symptoms (behavior problems), stress, and self-esteem (Pollard and Lee, 2003). Socio-cultural adjustment can be competence and mastery of behaviors, emotions and cognitions fitting to the host culture (Haslberger, 2005).

Despite the extensive literature focused on expatriate, spouse, and family adjustment, the study of adjustment in TCKs is still a relatively neglected area. In recent years, comprehensive reviews have been conducted on the concept of family systems in expatriate adjustment, transition programs, and identity development, as well as adult and college student TCK research (Sterle et al., 2018; Miller et al., 2020; Tan et al., 2021). While these are undoubtedly essential data, there still exists a gap in the literature for a review specifically focused on adjustment in TCKs. Additionally, many TCK adjustment studies were conducted through retrospective studies of childhood experiences (e.g., Decuyper et al., 2019) or by respondents other than the TCK themselves (Izumi and Gullón-Rivera, 2018). And although retrospective studies offer valuable insights into TCK adjustment, they also carry threats to internal and external validity (Tofthagen, 2012).

The current paper aims to fill this gap by providing a comprehensive systematic review synthesizing the available empirical evidence on adjustment in TCKs and focuses exclusively on findings during their relocation. To expand on current reviews, external indicators such as family functioning, stress, structure, social support, and demographic and mobility variables (such as age, gender, length and duration of expatriation, number of moves, home country, and host country) which predict adjustment were also included. We aim to understand factors related to TCK adjustment, highlight lacking research areas, and define areas of interest for future research.

## Methods

### Retrieval procedures

This review aimed to capture all available English-language peer-reviewed journal articles on the adjustment of school-aged TCKs aged 5 to 18 years during their international stay. We included all published articles from the beginning of time until December 2021 across nine electronic databases: APA Psychinfo, PSYNDEXplus Literature, and Audiovisual Media, ERIC, MEDLINE, web of science, Scopus, SocINDEX, and sociological abstracts (Supplementary Datasheet 1).

### Inclusion/exclusion criteria

The following eligibility criteria were set according to the PICO guidelines:

- Population: expatriate, third culture, cross-cultural, international, family relocation, sojourner, military, missionary, oil industry, oil patch, diplomat/Age sample: Kid, child, adolescent, youth, teen, family, student.
- Intervention: international relocation, measures are taken during the relocation.
- Comparison: some studies may use comparison groups (non-international/local). Both quantitative and qualitative studies were considered for inclusion.
- Outcome: wellbeing, adjustment, psychological adjustment, social adjustment, or adaptation.

The following conditions were set for inclusion:

- Participants aged between 5 and 17 years,
- Child/adolescent is the respondent,
- Child/adolescent has relocated internationally with their parent(s)/family,
- Measures have been taken during the international relocation,
- Expatriation is linked to parent/caregiver's employment,
- Adjustment is the primary outcome (including behavioral, affective, cognitive, academic, and socio-cultural determinants (Haslberger et al., 2014),
- Peer-reviewed published scientific articles.

We decided to focus on school-aged children as they are likely to interact within host communities, have developed language, friendships, and social references before the international move, and are therefore expected to be more affected by the stress from the relocation than younger children. We excluded late adolescents (19–21 years), tertiary students, and young adults as this population is likely to have moved away from their parents' homes to study and may need to adjust to circumstances other than the international move. We excluded papers that studied other expatriate populations (such as international students at the tertiary level, education migrants, high school exchange students, first and second-generation immigrants and migrants, child and adolescent adoptees, military deployment of a parent without family, and non-international relocation) as these populations have specific characteristics which may not entirely compare with traditional TCKs. Studies, where the respondent was not the child themself (teachers, parents, or retrospective studies from adult TCK) were excluded to limit the methodological biases which result from indirect measures. Other studies were excluded when the condition was not an international relocation (i.e., repatriation and returnees or domestic relocation). We excluded studies focusing on different themes than predictors and adjustment outcomes, such as testing the effect of specific programs. We also excluded non-empirical studies, for example, case reports, gray literature, reviews, unpublished work, theses, and commentaries. Studies were also excluded where the TCK data analysis was not separated from non-TCK groups.

### Screening and quality assessment

**Figure 1:**
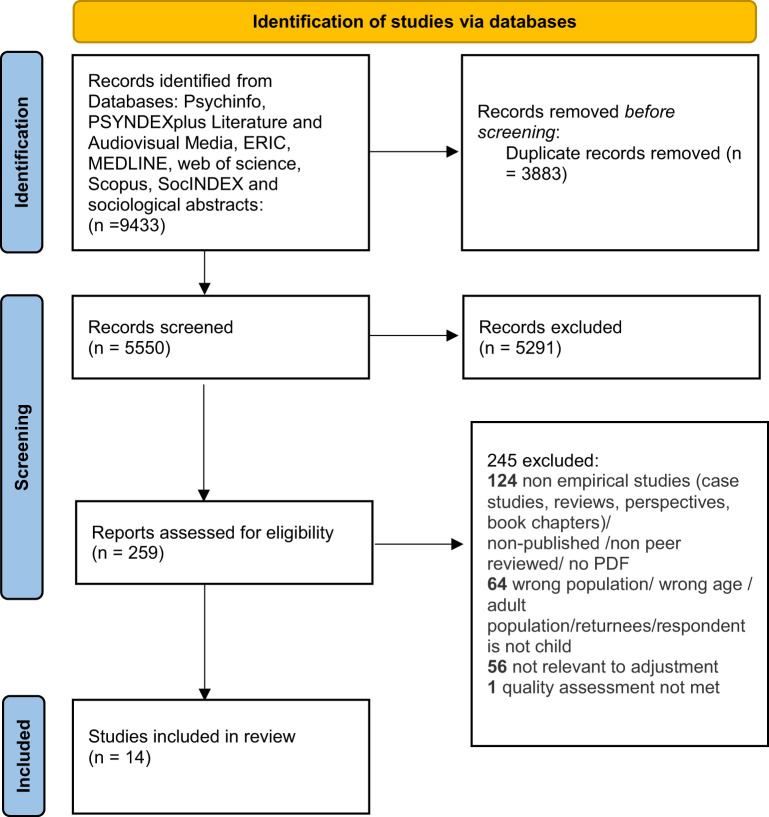
PRISMA 2020 flow diagram for new systematic reviews which included searches of databases and registers only. From Page et al. (2021). For more information, visit: http://www.prisma-statement.org/.

The online review management and screening tool Covidence was used to screen studies. Covidence is a web-based collaboration software platform that streamlines the production of systematic and other literature reviews (Covidence, 2021). The screening and selection of the papers based on title, abstract, full text, and quality control and extraction phases were conducted independently by 3 study team members (E.J., M.R. and Y.P.O.) and research assistants. For each paper, the quality of studies to extract was established independently by two study team members (E.J. and M.R. or E.J. and Y.P.O.) using Joanna Briggs Institute's critical appraisal tools (Critical-Appraisal-Tools, 2022). The 8-item checklist for analytical cross-sectional studies and the 10-item checklist for qualitative research was used^1^. Due to the small number of eligible studies, inclusion of each paper was based on consensus. Results from the process can be seen in the PRISMA chart presented in Figure 1 (Moher et al., 2009).

### Data abstraction and analysis

First, we defined a codebook that could be used to abstract findings in both quantitative and qualitative papers, and a content analysis of both quantitative and qualitative studies was conducted using Leximancer^2^ content analysis and concept mapping software. This automated analysis method offers an unbiased and objective data analysis (Smith and Humphreys, 2006; Angus et al., 2013). The software systematically extracts concepts from uploaded full-text studies and assembles the concepts into clusters according to their prominence and connectedness (Supplementary Image 1). Leximancer's yield was refined according to the researcher's knowledge of the selected studies. Next, we compared the clusters with the extracted theoretical references (Table 1) and deducted codes from these two abstractions. Last, the deducted codes were applied to Leximancer's ranked concept list (Table 2), allowing for details to be added to the codebook. This preliminary content analysis offers an overview of the higher-level themes and clusters of concepts explored in the selected research papers. The codebook was used as a grid to organize quantitative and qualitative study findings.

**Table 1:** Theories stated in extracted papers.

**Theoretical framework**	**Study number # (ID)**
**Third culture**:	#1 (Gerner et al., 1992), #2 (Ittel and Sisler, 2012), #5 (Morales, 2017), #7 (Lam and Selmer, 2004),
Useem (2001) and Pollock et al. (2010)	#11 (Langinier and Gaspoz, 2015), #13 (Mclachlan, 2007), #14 (Weeks et al., 2010)
**Socio cultural adjustment**	#2 (Ittel and Sisler, 2012), #3 (McKeering et al., 2021), #6 (Pittman and Bowen, 1994),
Sociocultural adjustment: Searle and Ward (1990),	#9 (Van Oudenhoven et al., 2007)
Ward and Kennedy (1999)
**Acculturation**	#1 (Gerner et al., 1992), #3 (McKeering et al., 2021), #9 (Van Oudenhoven et al., 2007)
Berry (1990); Berry et al. (2006)	
**Intercultural sensitivity**	#5 (Morales, 2017), #7 (Lam and Selmer, 2004), #8 (Straffon, 2003), #9 (Van Oudenhoven et al., 2007)
Bennett's Developmental Model of	#11 (Langinier and Gaspoz, 2015), #14 (Weeks et al., 2010)
Intercultural Sensitivity (DMIS) (Bennett, 1986, 1993)
Hofstede (1980, 2003)
Identity as a sociocultural construct: Hofstede (1980, 2003),
Valsinier (2007), place identity: Proshansky et al. (1983),
Twigger-Ross and Uzzell (1996), Kemph (1969)	#6 (Pittman and Bowen, 1994), #11 (Langinier and Gaspoz, 2015), #12 (Lijadi and Van Schalkwyk, 2017)
**Family stress and family systems**	#9 (Van Oudenhoven et al., 2007), #13 (Mclachlan, 2007)
e.g., the double ABC- X model, Patterson and McCubbin (1987)	
**Attachment**	#9 (Van Oudenhoven et al., 2007), #14 (Weeks et al., 2010)
Bowlby (1977)	
**Wellbeing, stress and coping**	#3 (McKeering et al., 2021), #10 (Vercruysse and Chandler, 1992), #12 (Lijadi and Van Schalkwyk, 2017)
Lazarus and Opton (1966), Lazarus and Folkman (1984), Moos (1984)
**Culture shock and learned helplessness**	#4 (Miyamoto and Kuhlman, 2001), #13 (Mclachlan, 2007)
Reinicke (1986), Oberg (1960), Toffler (1970)	
**Adult TCK models**	#14 (Weeks et al., 2010)
Black (1988), Shaffer and Harrison (2001) spouse
adjustment model

**Table 2:** Coded Leximancer ranked concept list.

**Concept**	**Count/relevance %**	**Designated code**
Family	434/100	Family
School	420/97	Academic/Sociocultural
Culture	393/91	Sociocultural
Relationship	375/86	Sociocultural
Adjustment	331/76	Adjustment
Intercultural	259/60	Sociocultural
Social	218/50	Sociocultural
Education	200/46	Family/Sociocultural
Parents	158/36	Family/Environment
Home	149/34	Family/Sociocultural
Stress	140/32	Psychological
Development	133/31	Psychological
Coping	132/30	Psychological
Engagement	132/30	Academic/Psychological
Work	131/30	Environment
Emotional	94/22	Psychological
Avoidance	86/20	Psychological
Identity	85/20	Psychological
Attachment	85/20	Psychological
Resilience	81/19	Psychological
Friends	78/18	Psychological/Sociocultural
Host	74/17	Sociocultural
Community	71/16	Sociocultural
Future	51/12	Sociocultural/Psychological

Subsequently, all 14 extracted studies were abstracted in Tables 3, 4 to the recommended strategy described in the Matrix Method (Garrard, 2020). Table 3 presents predictors of adjustment, extracted and organized into three categories using the predefined codes: psychological, academic, socio-cultural, family, and environmental. Then, following the Matrix Method, results from 10 quantitative (including one mixed methods) studies were abstracted to reveal significant findings. Only results reported as significant and with given correlation coefficients and *p*-values from each study were extracted (Table 4). Then, the four qualitative studies (including one mixed methods) were abstracted using a thematic synthesis approach, allowing recurring themes to be abstracted from qualitative data using thematic headings (Thomas and Harden, 2008).

**Table 3:** Descriptives and prominent findings in extracted studies.

**No**.	**Study ID**	**Country in which the study conducted**	**General theme/ background theory**	**Study design**	**Sample description**	**Age range**	**Mean age**	**Predictors (independent variables): factors in adjustment**	**Data analysis**	**Outcomes (dependent variables): measures of adjustment**	**Notes**
1	Gerner et al. (1992)	Egypt, Thailand, United States	Acculturation (Berry, 1990). characteristics of IM (internationally mobile) vs. non IM adolescents and characteristics of US IM adolescents vs. non US IM adolescents	Cross sectional quantitative, comparison groups of internationally mobile (IM) adolescents in Egypt and Thailand and non IM adolescents in USA/comparisons in between USA IM's and non USA IM's	Secondary school U.S. Internationally Mobile Adolescents (IM, n = 489 of which 125 from USA) international school in Thailand (ISB); and 365 (of which 147 from USA) international school in Egypt (CAC).) vs. U.S. Adolescents in the United States (Non-IM, n = 222) The internationally mobile sample: 34% from the United States, 26% from Asian countries, 17 % from European countries, 15 % from Middle Eastern countries, and 8 % from other nations:	Secondary school students	NS	Comparisons in between internationally mobile (IM) samples of Adolescents in international schools in Egypt and Thailand and a non-mobile samples of USA adolescents in a local school in the USA/comparisons in between US IM adolescents and non US IM adolescents in Thailand and Egypt	MANOVA, univariate F tests	The Internationally Mobile or Third-Culture Adolescent Questionnaire: Seven subscales: Family Relationship (10 items), Peer Relationship (8 items), Cultural Acceptance (6 items), Travel Orientation (7 items), Language Acceptance (5 items), Future Orientation (11 Items), and Stereotyping (10 items). + 13 items of biographical data.	
2	Ittel and Sisler (2012)	Germany	Factors of sociocultural adjustment in adolescent TCK	Cross sectional quantitative	Students from international schools in Berlin, Germany. Twenty-four nationalities	12–19 years	NS	Locus of Control Scale for Children (NS-LCOS) Sociocultural	Chi-Squared test	Adaptation Scale (SCAS)	
				with an average of 2.7 relocations.			Adaptation Scale (SCAS) Parent-Adolescent Communication Scale (PACS) Revised UCLA Loneliness Scale (R-UCLA) Multidimensional Scale of Perceived Social Support (MSPSS)		Socio Adaptation Scale (SCAS)	
3	McKeering et al. (2021)	Singapore	Psychological and socio cultural adjustment Searle and Ward, 1990; Berry, 1997; Ward and Kennedy, 1999; Ward and Rana-Deuba, 1999 and the PERMA framework (Seligman MEP, 2011)	Cross sectional quantitative	Students from years six to eight at an international school in Singapore (K-12) of 24 different nationalities, United Kingdom (40.4%), Australia (18%), China (6.2%), India (5.6%), and America and Japan (3.9% each), with 26 students (14.6%) identifying as having dual nationality.	10–14 years		Age, gender, time in country, time at school, number of moves. adjustment is measured through wellbeing, school engagement and resilience.	Descriptive and Correlational analyses ANOVA	Wellbeing, resilience and school engagement as measures of adjustment: The EPOCH Measure of Adolescent Wellbeing scale: engagement, Perseverance, Optimism, happiness The School Engagement Measure, MacArthur (SEM): behavioral, emotional and cognitive engagement The Brief Resilience Scale (BRS)	
4	Miyamoto and Kuhlman (2001)	United States	Predictors of culture shock, grades in school and anxiety over returning to Japan	Cross sectional quantitative	240 Japanese students living in the USA, 4th grade through 11th grade (8 different grade levels)	NS	NS	92 item scale self designed by researchers, 19 subscales: students' relationship with American friends, Japanese friends and teachers at both their American school and their Japanese school; students' relationship and communication with their parents; students' English and Japanese language skills; students' parents' English skills; dominant languages used for different activities; and level of culture shock and level of concern over returning to Japan.	Regression analysis	Level of culture shock and grades in American school were abstracted as relevant for analysis.	
5	Morales (2017)	China	Intercultural competence (Hofstede, 1980; Bennett, 1986) cultural patterns	Cross sectional study quantitative	TCK's from 48 different countries, 43 Koreans and 96 non Korean, in American-based, Middle States Accreditation (MSA) accredited school located in China	13–19 years	NS	Gender and nationality (Korean and non-Korean)	Descriptive statistics *T*-tests	ICSI The Intercultural Sensitivity Inventory (ICSI) (Bhawuk and Brislin, 1992) in English	
6	Pittman and Bowen (1994)	Multiple	Adolescent adjustment/ personal/ psychological, to the external environment, in relationships with parents	Cross sectional study quantitative	882 out of a larger survey addressing *n* = 458 in USA, *n*= 215 in Germany, *n* = 209 in the Pacific. One thousand one hundred and seventy adolescents from Air Force settlements around the world.	12–18 years	14.7 years	Adjustment factors: external: satisfaction with life in the air force, satisfaction with life in the current base location, whether the air force is a good fit for raising children; adjustment in relationships with parents; mobility factors (recency of relocation), location of move (within USA or overseas), residence (in or off the air base). Stressful situation was measured through "dissatisfaction with the rate of moving, dissatisfaction with treatment by locals, difficulty making new friends and difficulty leaving old friends.	Simultaneous multiple regression analysis/ bivariate correlations	Personal/ psychological adjustment: boredom, loneliness, fear and life satisfaction	Only the significance of relocation overseas vs. within USA was abstracted as separate analysis for international vs. non international relocation was not undertaken
								Resource factors: family support, friendship support. background factors: father's military rank, sex, race, age and family structure.			
7	Lam and Selmer (2004)	Britain and Hong Kong	Perceptions of “being international” (Useem, 2001)	Cross sectional quantitative	3 samples: British expatriate adolescents living in Hong Kong (BE), local HK adolescents living in HK (LHK) and local British Adolescents living in Britain (BB)	NS	BE 14.11 LHK 17.42 BB 14.66	Perceptions of being inter national: 32-item instrument designed by Hayden and Thompson (2000). International mobility preferences and consequences: 34-item instrument developed by Gerner et al. (1992).	Descriptive statistics, correlations, MANCOVA, ANCOVA, multiple range tests (*post-hoc* analysis)	Intergroup comparisons	
8	Straffon (2003)	South East Asia	Intercultural sensitivity (Bennett, 1986, 1993; Bhawuk and Brislin, 1992)	Cross sectional mixed methods	336 international school students from 43 different home countries	13–19 years	NS	Time spent in an interna tional school	Descriptive statistics, Pearson correlations (time and developmental stages)	IDI: Intercultural development inventory: 60 item self assessment, sub categories of ethnocentric stages: denial, defense, minimization/ethnorelative stages: Acceptance, adaptation and integration.	
9	Van Oudenhoven et al., 2007	37 different countries, although the majority resided in the Netherlands (13.5%), Singapore (13.5%), and France (12.5%)	Intercultural adjustment (Searle and Ward, 1990)	Cross sectional quantitative	104 expatriate children from 21 different home countries, living in 37 different countries, since 6 months to 15 years and who had moved countries between one and four times.	8–18years	(Mean/13.2, SD/2.41)	Family Character istics. The scales for family adaptability, cohesion, and communication drawn from the Family Inventories developed by Olson et al. (1986): Family adaptability, Family cohesion, Family communication/ Expatriate Work Characteristics. Expatriate work satisfaction seven-item scale derived from Ali et al. (2003), Support from the Company before and during the expatriation period derived from Ali et al. (2003)/Personality. The MPQ (van der Zee and van Oudenhoven, 2000) measures	Multiple regress ion analysis/ hierarchical analysis	Intercultural adjustment: COOP WONCA function cards (Nelson et al., 1990) to measure Psychological adjustment (quality of life) of expatriate children. Sociocultural adjustment, self made 8 item scale derived from Black's (1988) and De Leon and McPartlin (1995) with indicators of adjustment and satisfaction	
								Cultural Empathy, Open-mindedness, Social Initiative, Emotional Stability, and Flexibility/Attachment Styles. Attachment (Van Oudenhoven and Hofstra) measures Ambivalent, secure and dismissive avoidant attachment styles.			
10	Vercruysse and Chandler (1992)	Belgium	Coping strategies	Cross sectional quantitative	39 US adolescents and their parents living in Belgium since < 12 moths and attending various international schools	12–18 years	15.63	Background Information Data Sheet (sex, age, previous history of moves) Children Self-Concept Scale (PHCSCS, Piers and Harris, 1984), parent rated Stress Response Scale (Chandler and Shermis, 1990) measures the impact of stress on behavioral adjustment.	Means and standard deviations, Inferential statistics Ttest Point biserial and Pearson product-moment correlations	Coping responses inventory-Youth form (CRI-Y, Moos, 1990)	
11	Langinier and Gaspoz (2015)	Luxembourg	Identity (socio-cultural perspective) (Valsinier, 2007; Bruner, 2015)	Qualitative research	1) 5 teenagers 2) 10 expatriates	16–17	NS	Comprehensive approach: Interviews, informal discussions	A multilevel analysis based on intersectionality shows macro- and meso-level influences on the construction of nomadic identities	The authors differentiate three types of expression of nomadic identities based on distance from a culture, self perception and group identification/ cosmopolitan identity, transnational identity and anchor identity	
12	Lijadi and Van Schalkwyk (2017)	Macau and Hong-Kong	Place identity construction	CLET collage making/ qualitative interview	International school students	7–16 years	NS	CLET	CLET analysis	Themes: 1. Family, family rituals, and familiarity 2. My origin vs. countries where I have lived 3. Wishing for the ideal home 4. Expanding my network 5. Acquisitions and losses 6. Change as the only constant	
13	Mclachlan (2007)	UK	Family transience	Qualitative research	Students of a private international School in southern England and their parents. Forty-five families were involved	3 sub groups: 7–9 years, 10–12,	NS	20–40 min interviews with child participants, separately from their parents	Grounded theory or constant comparative method	Themes: guilty parents and grieving children; strengthening and restructuring; managing independence and cohesiveness; and parenting IM children	
14	Weeks et al. (2010). The adjustment of expatriate teenagers. *Personnel Review*.	Shanghai China	Teen adjustment (Shaffer and Harrison, 2001) compared to the model of expatriate spouse adjustment	Qualitative research	18 students at a private international school in Shanghai, China. Came to China for parents' employment, 14 out of 18 are from the USA.	14–19 years	NS	In-depth interviews	Coding of answers into 46 codes from 6 conceptual categories:	Themes: Individual factors: open-mindedness, freedom and academic success/Interpersonal relationship factors: friends, family and repatriation training/environmental factors: cultural differences and living all (adjustment)	

**Table 4:** Abstracted findings from quantitative studies.

**Category**	**Sub category**	**Factors of adjustment**	**Number of participants involved in finding (total participants in analysis)**	**Study # (study ID)**	**Gender**	**Direction of association: P, positive; N, negative**	**Orientation of outcome: P psychological adjustment; S, sociocultural adjustment; F, family adjustment, TCK, third culture; A, academic**	**Meaning of finding**
Demographic	Age	Age	178	#3 (McKeering et al., 2021)	MF	P	P	Younger children (10 years) are generally more happy and optimistic than 12–14 year old's (bigger risk for older TCK)/no difference for resilience
		Age	39	#10 (Vercruysse and Chandler, 1992)	MF	P	P	Older teenagers are more likely to use an approach coping strategy
	Gender	Gender	178	#3 (McKeering et al., 2021)	F	P	SC	Risk factor for student engagement: being male/no difference in for resilience or wellbeing
		Gender	39	#10 (Vercruysse and Chandler, 1992)	F	p	P	Females are more likely to use an approach coping strategy
	Nationality	Nationality non-US IM's vs. US IM's	272 (792)	#1 (Gerner et al., 1992)	MF	P	SC	IM adolescents from other countries rated themselves closer to their families, more interested in travel, more accepting of learning languages, and more inclined toward international careers than did US IM adolescents. Reversely, US IM adolescents rated more favorably on the stereotype scale than IM adolescents from other countries
	Mobility	Time at school	178	#3 (McKeering et al., 2021)	MF	p	P	Longer length of stay at school positively impacts wellbeing and resilience
		Time at international school	336	#8 (Straffon, 2003)	MF	P	SC	The longer students spend at an international school, the lower their scores in the denial and defense stages of intercultural sensitivity.
		Time in country	178	#3 (McKeering et al., 2021)	MF	p	P	Recent relocation to a new country affects student's ability to thrive (lower resilience/no effect on wellbeing)
Family factors	Family demographics	Number of younger siblings	240	#4 (Miyamoto and Kuhlman, 2001)	MF	P	SC	Fewer younger siblings is associated with better grades in American school
		Number of older siblings	240	#4 (Miyamoto and Kuhlman, 2001)	MF	p	SC	More older siblings is associated with higher levels of culture shock
	Family functioning	Family orientation	62	#7 (Lam and Selmer, 2004)	MF	p	F	Expatriate adolescents are closer to their family than their local counterparts in Hong Kong and GB
		Family cohesion	104	#9 (Van Oudenhoven et al., 2007)	MF	p	P+SC	Significant raw correlations with sociocultural adjustment and quality of life/family cohesion significantly predicts both quality of life and sociocultural adjustment in expatriate children
Environmental factors	Expatriate work	Expatriate parent work satisfaction	104	#9 (Van Oudenhoven et al., 2007)	MF	P	P + SC	Expatriate work satisfaction significantly predicts both quality of life and sociocultural adjustment in expatriate children
Psychological	Cognitive	Flexibility	62	#7 (Lam and Selmer, 2004)	MF	p	P	Expatriate adolescents are more flexible than their local counterparts in Hong Kong and GB
		Self efficacy	46	#2 (Ittel and Sisler, 2012)	MF	P	SC	TCKs who indicated high levels of general self-efficacy were significantly more likely to report fewer difficulties in socio-cultural adaptation
		Stereotyping	147 (494)	#1 (Gerner et al., 1992)	MF	P	SC	US adolescents in an international school in Egypt were significantly more accepting of other cultures (lower level of stereotypical judgement than their peers living in the US and in the International school in Thailand. This single effect is specific to expatriate adolescents living in Egypt.
	Personality	Open-mindedness toward other cultures	62	#7 (Lam and Selmer, 2004)	MF	p	P	Expatriate adolescents are more open minded toward other cultures than their local counterparts in Hong Kong and GB
	Attachment	Ambivalent attachment style	104	#9 (Van Oudenhoven et al., 2007)	MF	N	P	Ambivalent attachment style significantly hinders both quality of life and sociocultural adjustment in expatriate children/moderation effect ambivalent attachment style interacted significantly with expatriate work satisfaction in its influence on quality of life
	Emotional	Emotional stability	104	#9 (Van Oudenhoven et al., 2007)	MF	P	P + SC	Emotional stability significantly predicts both quality of life and sociocultural adjustment in expatriate children/interaction effect (moderation) with expatriate work, family cohesion and family communication on sociocultural adjustment and quality of life
		Repatriation anxiety (here about returning to japan)	240	#4 (Miyamoto and Kuhlman, 2001)	MF	P	SC	Less anxiety about returning to japan predicts better grades in the American school.
	Social	Respect and tolerance of others	63	#7 (Lam and Selmer, 2004)	MF	p	P + TCK	Expatriate adolescents have more respect and tolerance of others than their local counterparts in Hong Kong and GB
	Identity	Own cultural identity	62	#7 (Lam and Selmer, 2004)	MF	p	TCK	Expatriate adolescents have their own cultural identity which differs significantly from that of their local counterparts in Hong Kong and GB
Sociocultural factors	Relationships	Perceived relationships with teachers (from international location)	240	#4 (Miyamoto and Kuhlman, 2001)	MF	P	SC	Better perceived relationships with American school teachers predicts less culture shock
		Perceived peer relationships	46	#2 (Ittel and Sisler, 2012)	MF	N	SC	This negative relationship speaks for a buffering potential of close friendships on socio cultural adaptation
		Perceived relationship with local friends	240	#4 (Miyamoto and Kuhlman, 2001)	MF	P	SC	Better perceived relationships with American friends predicts less culture shock
		Usage of internet to connect with friends/family from around the world	46	#2 (Ittel and Sisler, 2012)	MF	P	SC	TCKs who frequently utilize the world-wide web and make use of internet communities of other children and adolescents with similar multiple
								migration backgrounds to connect and maintain contacts are less likely to have difficulties in the adaptation process
	Culture	Cultural acceptance	272 (494)	#1 (Gerner et al., 1992)	MF	P	SC + TCK	US adolescents in international schools in Thailand and Egypt are significantly more culturally accepting than their peers living in the US. This effect is due to the International mobility factor rather than location because it affects both internationally mobile groups.
	International mobility	International career preference	62	#7 (Lam and Selmer, 2004)	MF	p	TCK	Expatriate adolescents will prefer an international career above their local counterparts in Hong Kong and GB
		International travel preference	62	#7 (Lam and Selmer, 2004)	MF	p	TCK	Expatriate adolescents will prefer to travel above their local counterparts in Hong Kong and GB
		Travel orientation	272 (494)	#1 (Gerner et al., 1992)	MF	P	TCK	US adolescents in international schools in Thailand and Egypt are significantly more keen on traveling than their peers living in the US. This effect is due to the International mobility factor rather than location because it affects both internationally mobile groups.
		Settling down preference	62	#7 (Lam and Selmer, 2004)	MF	n	TCK	Expatriate adolescents are less keen on settling down in one place than their local counterparts in Hong Kong and GB
		Future orientation (international)	272 (494)	#1 (Gerner et al., 1992)	MF	P	TCK	US adolescents in international schools in Thailand and Egypt are significantly more orientated toward living and working abroad in the future than their peers living in the US. This effect is due to the International mobility factor rather than location because it affects both internationally mobile groups.
	Language	Language proficiency level self reported (English by Japanese students)	240	#4 (Miyamoto and Kuhlman, 2001)	MF	P	SC	Better perceived proficiency in English positively predicts better grades in American school
		Foreign language interest	62	#7 (Lam and Selmer, 2004)	MF	p	SC + TCK	Expatriate adolescents have more interest in learning foreign languages than their local counterparts in Hong Kong and GB
		Level of motivation for maintaining Japanese language skills	240	#4 (Miyamoto and Kuhlman, 2001)	MF	p	SC	Higher levels of motivation for maintaining home language (Japanese) reduces culture shock
		Language acceptance	272 (494)	#1 (Gerner et al., 1992)	MF	P	SC + TCK	US adolescents in international schools in Thailand and Egypt are significantly more interested in other languages than their peers living in the US. This effect is due to the International mobility factor rather than location because it affects both internationally mobile groups.
	Academic factors	Grades in Japanese supplementary school	240	#4 (Miyamoto and Kuhlman, 2001)	MF	P	SC + A	Better grades in Japanese supplementary school significantly predict grades in American school
		Perceived ease of completing homework	240	#4 (Miyamoto and Kuhlman, 2001)	MF	p	SC + A	Better perceived ease with completing homework from school in international location significantly decreases culture shock

### Thematic and conceptual extraction

Theoretical frameworks and references were extracted from the included studies and organized into categories, as shown in Table 1. Concurrently, researchers extracted clusters from the Leximancer content analysis: the concept map (Supplementary Image 1) shows four clusters of themes where family, stress, and coping (labeled “psychological”); school and culture (labeled “'socio-cultural”); and engagement (labeled “environment”) stand out. The links within these clusters show the most frequently associated themes, allowing the authors to label each cluster accurately. We used the clusters and extracted theoretical references to deduct the following codes: environmental, family, socio-cultural and psychological. Table 2 shows the ranked concept list from Leximancer, where the above codes have been applied to each concept, allowing researchers to refine the labels. The final codebook is presented below.

Predictors:

- Demographic and environmental factors: age, gender, nationality, mobility, and parent work.
- Family factors: family support, family functioning, and parental stress.
- Psychological factors: cognitive, personality, attachment, emotion, behavior, social skills, and identity.
- Socio-cultural factors, friendships, home, and culture, including intercultural sensitivity, acculturation, language, and school.

Outcomes:

- Psychological adjustment includes wellbeing, stress, and coping.
- Socio-cultural adjustment includes culture shock and acculturative stress.
- Third culture identity includes place identity and specific traits.

A thematic synthesis of the qualitative studies was undertaken following three stages (Thomas and Harden, 2008): (1) line-by-line coding of study findings and direct quotations using the predefined codebook, (2) abstracting the themes and findings from the qualitative studies, then (3) grouping coded findings to generate analytical themes across studies. All interviews addressed child and adolescent TCKs; one study included images as an addition to the interviews, and one included parents in separate interviews. Results from family interviews were only considered when it was clear that the child respondent originated a comment or idea.

## Results

### Preliminary analysis of studies

Table 5 presents studies ordered by continents, 5 year-periods, and journal types. The studies are evenly distributed over the past two decades and have been conducted primarily in Asia and Europe, whereas three were conducted across different continents. Studies were published in 13 psychology, development, education, society, intercultural, and human resources journals. Nine studies were quantitative, one used a mixed-methods design, and four were qualitative.

Table 5Study characteristics.

**Table:** 

**Studies per continents**				
	**Europe**	**US**	**Asia**	**Cross-continent**
				#1: US/Asia/Africa
				#6: Europe/US/Pacific
				#7: Europe/Asia
Study # (ID)	#2 (Ittel and Sisler, 2012), #9 (Van Oudenhoven et al., 2007), #10 (Vercruysse and Chandler, 1992), #11 (Langinier and Gaspoz, 2015), #13 (Mclachlan, 2007)	#4 (Miyamoto and Kuhlman, 2001)	#3 (McKeering et al., 2021), #5 (Morales, 2017), #8 (Straffon, 2003), #14 (Weeks et al., 2010), #12 (Lijadi and Van Schalkwyk, 2017)	#1 (Gerner et al., 1992), #6 (Pittman and Bowen, 1994), #7 (Lam and Selmer, 2004)
*n*	5	1	5	3
%	36%	7%	36%	21%

**Table:** 

**Studies per 5 y-periods since 1992**					
**Year**	**1992–1997**	**1998–2003**	**2004–2009**	**2010–2015**	**2016–2021**
Study number	#1 (Gerner et al., 1992), #6 (Pittman and Bowen, 1994), #10 (Vercruysse and Chandler, 1992)	#4 (Miyamoto and Kuhlman, 2001), #8 (Straffon, 2003)	#7 (Lam and Selmer, 2004), #9 (Van Oudenhoven et al., 2007), #13 (Mclachlan, 2007)	#2 (Ittel and Sisler, 2012), #11 (Langinier and Gaspoz, 2015), #14 (Weeks et al., 2010)	#3 (McKeering et al., 2021), #5 (Morales, 2017), #12 (Lijadi and Van Schalkwyk, 2017)
*n*	3	2	3	3	3
%	21.43%	14.28%	21.43%	21.43%	21.43%

**Table:** 

**Journals**												
**Psychology**		**Development**			**Education**			**Intercultural**		**Human resources**		
Journal of School Psychology	Anxiety, Stress, & Coping	Journal of Childhood and Adolescence Research	Journal of Adolescence	Youth & Society	Journal of Research in International Education	Journal of International Education Research	Geoforum	International journal of intercultural relations	International Journal of Intercultural Relations	Equality, Diversity and Inclusion: An International Journal	Career Development International	Personnel Review
#1 (Gerner et al., 1992)	#9 (Van Oudenhoven et al., 2007)	#2 (Ittel and Sisler, 2012)	#10 (Vercruysse and Chandler, 1992)	#6 (Pittman and Bowen, 1994)	#3 (McKeering et al., 2021), #13 (Mclachlan, 2007)	#5 (Morales, 2017)	#12 (Lijadi and Van Schalkwyk, 2017)	#8 (Straffon, 2003)	#4 (Miyamoto and Kuhlman, 2001)	#11 (Langinier and Gaspoz, 2015)	#7 (Lam and Selmer, 2004)	#14 (Weeks et al., 2010)

### Factors of adjustment in quantitative studies

The 10 extracted quantitative studies' findings were abstracted and presented in Table 4 (Garrard, 2020). Significant results in each study are labeled according to the study number in Table 3 and the predefined codebook. Non-significant and null findings, correlations, and statistical weights can be found in Supplementary Datasheet 2. All 10 studies utilized surveys, out of which three were designed by the researchers (Pittman and Bowen, 1994; Miyamoto and Kuhlman, 2001; Straffon, 2003). One study used a mixed-methods approach. Comparison groups with local (non-international children/adolescents) were used in 4 out of the 10 studies (Gerner et al., 1992; Pittman and Bowen, 1994; Lam and Selmer, 2004; Morales, 2017).

#### Demographic variables

Ages ranged from 7 to 19 years, and samples included male and female participants of similar proportions. Sample sizes ranged from 39 to 272 in the TCK groups. Two studies found age to influence adjustment: notably, older adolescents were more likely to struggle with adjustment, and older teenagers used a more elaborate (approach vs. avoidance) coping strategy (Vercruysse and Chandler, 1992; McKeering et al., 2021) (*n* = 217). Gender was found to influence adjustment in two studies, with male students being less engaged at school and female TCK using a more elaborate (approach vs. avoidance) coping strategy (Vercruysse and Chandler, 1992; McKeering et al., 2021) (*n* = 217). Length of stay in the current setting positively predicted adjustment outcomes in 2 studies (Straffon, 2003; McKeering et al., 2021) (*n* = 692).

#### Family variables

The family was investigated in two studies, with TCK reportedly feeling closer to their families and family cohesion positively influencing adjustment (Lam and Selmer, 2004) (*n* = 62), (Van Oudenhoven et al., 2007) (*n* = 166).

#### Psychological variables

For personality traits, TCK were more open-minded, respectful, and flexible toward other cultures compared to their local counterparts (Gerner et al., 1992) (*n* = 147); (Lam and Selmer, 2004) (*n* = 62). Factors that improve adjustment outcomes are emotional stability (Van Oudenhoven et al., 2007) (*n* = 104) and self-efficacy (Ittel and Sisler, 2012) (*n* = 46). Factors that hinder adjustment outcomes are ambivalent attachment style (Van Oudenhoven et al., 2007) (*n* = 104) and repatriation anxiety (Miyamoto and Kuhlman, 2001) (*n* = 240).

#### Sociocultural variables

The perceived quality of social relationships with teachers, local friends (Ittel and Sisler, 2012) (*n* = 46), and those left behind (Miyamoto and Kuhlman, 2001) (*n* = 240) predict better adjustment. TCK were more interested in learning languages (Lam and Selmer, 2004) (*n* = 62), traveling (Gerner et al., 1992; Lam and Selmer, 2004) (*n* = 334), seeking a future abroad (Lam and Selmer, 2004) (*n* = 62) than their local peers. These findings are supported by measuring a distinct cultural identity (Lam and Selmer, 2004) (*n* = 62). Local language proficiency is shown to play a role in enhancing adjustment (Miyamoto and Kuhlman, 2001) (*n* = 240), whereas maintaining interest in “home language” reduces culture shock. TCK were generally more interested in language acquisition than their local counterparts (Gerner et al., 1992) (*n* = 272).

#### Orientation of outcomes

Psychological adjustment was explored through 12 findings, socio-cultural outcomes were explored through 22 findings, and the third culture was examined in 10 findings. In three cases, the same variable influenced socio-cultural and psychological adjustment. In one case, a psychological adjustment outcome was associated with a third culture trait. Three socio-cultural adjustment outcomes were associated with third culture traits.

### Factors of adjustment in qualitative studies

#### Environmental factors

##### Context

Stability is an important protective factor to support adjustment when the context changes and can be found in immediate family rituals and maintained connections with extended family and friends (Mclachlan, 2007; Lijadi and Van Schalkwyk, 2017).

##### Time

Time spent abroad and in contact with diverse communities enhances an ethno-relative worldview and supports better acceptance of other cultures (Straffon, 2003).

##### Repatriation/high mobility

Fears of repatriation or frequent moves and lack of permanence may increase stress and hinder adjustment (Weeks et al., 2010).

#### Family factors

Child interviewees report increased family closeness through meetings, discussions, and meals, to supplement the lack of an extended family or other extensions (Mclachlan, 2007). Family closeness is a sensitive topic, bearing possibilities to support each other and the risk of a closeness that might raise tensions and limit autonomy. Being involved in the family's decision to move (communication) generally contributes to the child/teen's agreeableness with the move (Mclachlan, 2007). Family relationships contribute to a sense of safety, providing comfort and continuity (belonging and direction) during the initial adjustment phase and helping to reduce stress from situations when they arise. Family members and the rituals of family life and the objects associated with them provide a sense of continuity, replacing the physical concept of home. Connectedness with extended family and grandparents contributes to a sense of home and stability (Lijadi and Van Schalkwyk, 2017).

#### Psychological factors

##### Personality

Child personality is raised as a determining factor, and agreeableness toward the move creates an opportunity to embrace change (Mclachlan, 2007). Open-mindedness is critical for making friends and adopting a worldview, including in international schools where students have diverse cultures and origins (Weeks et al., 2010).

##### Emotion

Grief from loss and longing can be related to places, memories, objects, perceived changes in family roles and responsibilities, or even a lost psychological state (Lijadi and Van Schalkwyk, 2017).

TCKs describe mixed emotions of excitement, disappointment, and anticipation as they repeatedly adjust to change.

##### Identity

Adolescence is a susceptible age for a move. Integrating the multiplicity of values of the various systems to which TCKs are exposed, as well as their differences in being multi-lingual, multicultural, and aware of the diversity of the world, creates an extra challenge in the identity formation process (Langinier and Gaspoz, 2015; Lijadi and Van Schalkwyk, 2017). Identification with a particular place, culture, and community call for a specific model to be defined for TCKs, which differs from identity construction and identification in non-TCKs (Langinier and Gaspoz, 2015; Lijadi and Van Schalkwyk, 2017). “TCK identity” becomes an entity within which TCKs are more inclined toward each other. Langinier and Gaspoz (2015) develop the idea of three expressions of identity (cosmopolitan, transnational, and anchor) dependent on identifications to national or international communities and where TCKs experience and social background influence the development of one or the other identity (Langinier and Gaspoz, 2015).

#### Socio-cultural factors

##### Friendships

Loss of friends in international settings is a commonly raised issue; TCKs must grieve friends from home and face the departures of friends and teachers in international schools (Weeks et al., 2010; Lijadi and Van Schalkwyk, 2017). TCKs report casual friendships rather than close ones, which could be their way of dealing with repeated loss or a bias in reporting and hiding underlying grief difficulties (Mclachlan, 2007). Difficulties entering already formed friend groups or communicating with peers can be a significant deterrent for adjustment and integration, whereas identifying and making friends they can identify with is raised by teens as the most important factor of overall adjustment (Weeks et al., 2010).

##### Home

Children maintain a bond with their passport country(ies) and the different places they have lived, which provides a sense of attachment. Positive feelings and memories during times spent in these places contribute to the sense of connectedness to a place (Lijadi and Van Schalkwyk, 2017). A challenge in adjustment arises when there is too big a gap between an idealized place and life challenges in that place.

##### Culture

Learning about a new culture can mean more freedom for adolescents, exploration, and easier access to drugs and alcohol in the host culture. These are mentioned as either contributing to autonomy and identity construction or creating a riskier environment and hindering the adjustment process (Weeks et al., 2010). Teenagers in international schools may feel at home in their host country without assimilating or integrating into their host country's culture. Friendships and the school environment majorly contribute to the sense of homeliness. Teenagers socializing within their international communities may preserve a surface-level interaction and understanding of their host culture (Weeks et al., 2010). Housing and comfort are positively related to adjustment and feeling at home.

##### Language

TCKs in international schools do not consider language a primary factor in their adjustment. Host language fluency is placed behind friendships and family relationships, as they are not dependent on the host culture to make friends or integrate. However, language acquisition has the potential to enhance the TCK's familiarity with their surroundings (Weeks et al., 2010). Home country language fluency is often maintained as a thread to home or to facilitate potential repatriation (Lijadi and Van Schalkwyk, 2017).

## Discussion

This systematic review is the first to synthesize the available data on factors that influence adjustment in child and adolescent TCKs during their international experiences. It also offers the reader an organized overview of empirical evidence on factors influencing TCK adjustment. Only 14 studies met our eligibility criteria despite screening across eight electronic databases. This yield speaks for the limited empirical evidence on child and adolescent TCK adjustment. Findings from this systematic review point toward gaps in the knowledge about the particular needs and traits that define child and adolescent TCK.

### Factors in TCK adjustment

Both quantitative and qualitative studies find specific variables contributing to TCK functioning and adjustment. Categories of factors that are shown to influence adjustment in TCK include demographics (age, gender, time/mobility, cultural background), family (demographics, functioning, support, and cohesion), environmental (expatriate work), psychological (cognitive and personality traits, attachment style, emotion, empathy, identity) and socio-cultural (relationships, friends, in particular, culture, language, school, and international mobility factors). Each factor contributes to or hinders psychological and socio-cultural adjustment or contributes to forming a specific third culture. Although studies have measured various factors and pinpointed the effects of these factors on TCK adjustment, there is a lack of cohesion between variables and outcomes. Only peer relationships on the outcome of socio-cultural adjustment and travel preference on the outcome of a third culture were tested twice. The interest in languages on the outcome of socio-cultural adjustment was tested only three times. This is in contrast to adult expatriate research showing that language plays a key role in adjustment (for example Selmer, 2006). This could be due to the limited number of studies in our review. However, it is also possible that the selected studies explore expatriate children in international schools who are not as exposed to the host culture and language as their adult counterparts, as the medium of teaching is often English. Clearly, more research on the role of language in TCK adjustment is needed.

In general, more research is needed to assert these findings, which remain scarce in number and sample size. Moreover, future models may include mediation and moderation factors. The coding categories deducted for this systematic review may continue to be used as a guide for future studies.

#### Demographics and environmental factors

This systematic review shows that demographic and mobility factors have been considered across four studies in total. Only one study compared two international locations but found mobility overrides the actual location (Gerner et al., 1992). Another single study compared TCK with local peers. Efforts must be made to refine sample characteristics using demographic variables (Aderi et al., 2013). Samples of various age categories and family structures will further define the contribution of these demographic variables. More research is needed where comparison groups could help understand the influence of cultures and nationalities on adjustment.

#### Family factors

Qualitative studies have expanded upon the family factors involved in adjustment, including cohesion, parenting, and family rituals. Only two studies measured family characteristics, parent relationships, and family demographic variables in quantitative designs (Pittman and Bowen, 1994; Van Oudenhoven et al., 2007). More quantitative studies, including measures of family functioning, family cohesion, parenting, and family demographics, will assert these findings, as suggested by Sterle et al. (2018).

#### Psychological factors

Psychological factors are particularly under-investigated, although shown to largely contribute to wellbeing and adjustment (Arslan, 2019). Potential mediation and moderation effects, particularly the interaction between third culture and psychosocial adjustment, as well as family functioning and psychosocial adjustment, need to be investigated (Zeng et al., 2022).

#### Toward a broader model of adjustment

Future research may refine our understanding of TCK adjustment by devising and testing more inclusive models and multiple trajectories in adjustment (Haslberger et al., 2014; Hirai et al., 2015; Mesidor and Sly, 2016). The classification proposed in this review includes categories of environmental, family, psychological and socio-cultural factors as a general frame for understanding the interactions between factors and outcomes of TCK adjustment and may serve as a guide for future studies and the foundation for a model of TCK adjustment.

### Defining and measuring adjustment

Extracted studies are scattered across the areas of psychology, development, education, human resources, and intercultural sciences. There is also diversity in the scope of theoretical references used to frame the research. Psychological adjustment may be linked to attachment theory, coping, identity, social identity, place identity concepts, and notions of stress and wellbeing. Socio-cultural adjustment may refer to Berry's acculturation theory, Bennett's intercultural sensitivity model, or notions of culture shock (Berry, 1980; Berry et al., 2006; Bennett and Hammer, 2017).

In some cases, adult adjustment models are used as models of child adjustment. Two studies also referenced family models (family stress and family functioning) (Pittman and Bowen, 1994; Van Oudenhoven et al., 2007). Theories used to frame research on TCK primarily target a specific model and explore either family, culture, identity, or psychological traits. The diverse theories and research found in this systematic review suggest that distinctive models may not reflect the entire process of TCK adjustment. More likely, adjustment at a point in time but also over time and identity outcomes are interconnected with psychological, socio-cultural, and environmental factors. As proposed for adult expatriates, a model reflecting these interrelations is needed for TCK (Haslberger et al., 2014).

### Defining the TCK sample

The theoretical complexity continues with diverse samples falling under the generic understanding of the meaning of TCK: a reflection of this diversity can be read through the multiple terms (e.g., military, internationally mobile, TCK, expatriate) used across studies to refer to the particular population. Half of the studies in this review referred to Pollok and Van Reken's or Useem's definition of TCK (Useem and Useem, 1967; Pollock et al., 2010). The lack of cohesion in the definition of the sample itself is an insight into the diversity of the specific experiences associated with particular reasons underlying the international relocation. Another fundamental challenge for researching this population lies in the diverse nationalities of origin and relocation, age groups, duration of stay, types of schools, and family structures contributing to the variation in adjustment. One example of sampling difficulty can be found in comparing the following studies: the case of exploring culture shock in Japanese students adjusting to the U.S. and the other studying intercultural adjustment in TCK from 21 different home countries living in 37 different host countries (Miyamoto and Kuhlman, 2001; Van Oudenhoven et al., 2007). As the populations are so diverse, each study may only apply to a particular cultural sample and may not be generalizable to other TCK groups. To conclude, we suggest that the ecological complexity reflected in this systematic review may be better approached through the lens of complex systems, which can account for individual, contextual and cultural interactions (Brown and Goetz, 1987; Schwartz et al., 2010).

#### Study designs and measures

Studies included in this review have used a variety of measures, some designed for the study by the researchers, some based on pre-existing scales, and some using validated scales with normative information for a general population. Normative studies using validated scales could help create a standard for TCK, which would contribute to a better understanding of the outcomes of future quantitative studies. Reproducing studies using a particular scale would help assert the findings from an ecological standpoint and increase the consistency of results. Lastly, no study used a longitudinal design despite the specific sensitivity of time measured (as a predictor of mobility) in two of the presented studies (Fisher and Shaw, 1994; Straffon, 2003; Pritchard et al., 2007; McKeering et al., 2021). Future cohort studies, particularly those using a longitudinal design, as has been done with adult and college student expatriate samples, would reinforce findings from the cross-sectional studies available this far (Fisher and Shaw, 1994; Pritchard et al., 2007).

### Limitations

Although this study has the merit of synthesizing available data on a clearly defined ecological sample, it has several limitations. First, the restrictive criteria for inclusion meant that only a small number of papers were included and studies with multiple informants, such as parents and teachers, were excluded. Other unpublished or pilot studies may contribute to TCK adjustment but were not included in this study to ensure the strong validity of our findings. Further, the abstracted results from quantitative studies were not included in a meta-analysis due to the heterogeneity of predictors and outcomes and the variety of analyses used and reported.

### Conclusions

This review highlights the complexity of defining the TCK sample, the diversity of internal and external factors contributing to TCK adjustment, and the formation of a “third culture.” Because of this, the network of selected studies stands out as heterogeneous and difficult to analyze. To better assess the needs and characteristics of TCK, efforts can be made to improve the ecological validity of study samples and to consider adjustment within an inclusive multi-faceted model or through the lens of complex adaptive systems (Arrow et al., 2000; Nettle et al., 2013; Haslberger et al., 2014; Theodore and Bracken, 2020). More research is needed on TCKs at the time of the relocation, and over time and more effort can be made to improve the methodological quality of measures.

## Data availability statement

The original contributions presented in the study are included in the article/Supplementary material, further inquiries can be directed to the corresponding author/s.

## Author contributions

EJ conceived the structure of the manuscript. EJ, MR, and YO reviewed the papers. EJ and MR drafted the manuscript. All authors edited the manuscript and read and approved the final manuscript.

## Funding

Funding for this project was provided by the Division of Clinical Psychology and Psychotherapy, Faculty of Psychology, University of Basel.

## Conflict of interest

The authors declare that the research was conducted in the absence of any commercial or financial relationships that could be construed as a potential conflict of interest.

## Publisher's note

All claims expressed in this article are solely those of the authors and do not necessarily represent those of their affiliated organizations, or those of the publisher, the editors and the reviewers. Any product that may be evaluated in this article, or claim that may be made by its manufacturer, is not guaranteed or endorsed by the publisher.

## Footnotes

1 https://jbi.global/critical-appraisal-tools

2 https://www.leximancer.com
