# Supplementary material for: Adjustment in third culture kids: A systematic review of literature
Source: Front Psychol. 2022 Nov 28;13:939044. doi: 10.3389/fpsyg.2022.939044 (PMC9743971; doi:10.3389/fpsyg.2022.939044)
Supplement: Supplementary file 1 [file Data_Sheet_1.docx]

************************************

APA PsycInfo (Ovid, Advanced Search)

************************************

((Expat* or "third culture" or "cross cultural" or international or "family relocation" or sojourn* or military or missionary or "oil industry" or "oil patch" or diplomat*) adj4 (kid* or child* or adolescent* or youth or teen* or famil* or student*)).ti,ab,id. and (("well-being" or adjust* or adapt*).ti,ab,id. or (well-being or adjustment or adaptation or "Resilience (Psychological)").sh. or ("Adaptation, Psychological" or "Social Adjustment").mh.)

Results: 2525

********************************************************************

PSYNDEXplus Literature and Audiovisual Media (Ovid, Advanced Search)

********************************************************************

((Expat* or "third culture" or "cross cultural" or international or "family relocation" or sojourn* or military or missionary or "oil industry" or "oil patch" or diplomat*) ADJ4 (kid* or child* or adolescent* or youth or teen* or famil* or student*)).ti,ab,id AND (("well-being" OR adjust* OR adapt*).ti,ab,id OR (well-being OR adjustment OR adaptation).sh)

Results: 51

****************************

ERIC (Ovid, Advanced Search)

****************************

((Expat* or "third culture" or "cross cultural" or international or "family relocation" or sojourn* or military or missionary or "oil industry" or "oil patch" or diplomat*) ADJ4 (kid* or child* or adolescent* or youth or teen* or famil* or student*)).ti,ab AND (("well-being" OR adjust* OR adapt*).ti,ab OR ("Well Being" OR "Social Adjustment").sh)

Results: 858

************************************

MEDLINE (All, Ovid, Advanced Search)

************************************

((Expat* or "third culture" or "cross cultural" or international or "family relocation" or sojourn* or military or missionary or "oil industry" or "oil patch" or diplomat*) ADJ4 (kid* or child* or adolescent* or youth or teen* or famil* or student*)).ti,ab,kw AND (("well-being" OR adjust* OR adapt*).ti,ab,kw OR ("Adaptation, Psychological" OR "Social Adjustment").sh)

Results: 1437

***************************************

Web of Science (Core Collection, Topic)

***************************************

TS=(((Expat* or "third culture" or "cross cultural" or international or "family relocation" or sojourn* or military or missionary or "oil industry" or "oil patch" or diplomat*) NEAR/3 (kid* or child* or adolescent* or youth or teen* or famil* or student*)) AND ("well-being" OR adjust* OR adapt*))

Results = 3855

*****************

Scopus (Advanced)

*****************

(TITLE-ABS-KEY ( (Expat* OR "third culture" OR "cross cultural" OR international OR "family relocation" OR sojourn* OR military OR missionary OR "oil industry" OR "oil patch" OR diplomat*) W/4 (kid* OR child* OR adolescent* OR youth OR teen* OR famil* OR student*) ) ) AND (TITLE-ABS-KEY ( "wellbeing"OR adjust* OR adapt*) OR INDEXTERMS( "Adaptation, Psychological" OR "Social Adjustment" OR wellbeing OR adjustment OR adaptation) )

Results: 4480

*********************************

SocINDEX (EBSCO, Advanced Search)

*********************************

( ( (TI ((Expat* OR "third culture" OR "cross cultural" OR international OR "family relocation" OR sojourn* OR military OR missionary OR "oil industry" OR "oil patch" OR diplomat*) N3 (kid* OR child* OR adolescent* OR youth OR teen* OR famil* OR student*)) OR (AB ((Expat* OR "third culture" OR "cross cultural" OR international OR "family relocation" OR sojourn* OR military OR missionary OR "oil industry" OR "oil patch" OR diplomat*) N3 (kid* OR child* OR adolescent* OR youth OR teen* OR famil* OR student*)) OR (KW ((Expat* OR "third culture" OR "cross cultural" OR international OR "family relocation" OR sojourn* OR military OR missionary OR "oil industry" OR "oil patch" OR diplomat*) N3 (kid* OR child* OR adolescent* OR youth OR teen* OR famil* OR student*)) ) ) AND ( ( (TI ("well-being" OR adjust* OR adapt*) OR AB ("well-being" OR adjust* OR adapt*) OR KW ("well-being" OR adjust* OR adapt*) OR DE ("WELL-being" OR "SUBJECTIVE well-being (Psychology)" OR "SOCIAL adjustment" OR "ADAPTABILITY (Psychology)" OR "PSYCHOLOGICAL adaptation")) ) )

Results: 2588

***********************************************

Sociological Abstracts (ProQuest, Command Line)

***********************************************

(((TI("well-being" OR adjust* OR adapt*) OR AB("wellbeing" OR adjust* OR adapt*) OR IF("well-being" OR adjust* OR adapt*) OR SU("Well Being" OR Adjustment)) AND ((TI((Expat* OR "third culture" OR "cross cultural" OR international OR "family relocation" OR sojourn* OR military OR missionary OR "oil industry" OR "oil patch" OR diplomat*) NEAR/4 (kid* OR child* OR adolescent* OR youth OR teen* OR famil* OR student*))) OR (AB((Expat* OR "third culture" OR "cross cultural" OR international OR "family relocation" OR sojourn* OR military OR missionary OR "oil industry" OR "oil patch" OR diplomat*) NEAR/4 (kid* OR child* OR adolescent* OR youth OR teen* OR famil* OR student*))) OR (IF((Expat* OR "third culture" OR "cross cultural" OR international OR "family relocation" OR sojourn* OR military OR missionary OR "oil industry" OR "oil patch" OR diplomat*) NEAR/4 (kid* OR child* OR adolescent* OR youth OR teen* OR famil* OR student*))))) AND PEER(yes)) AND PEER(yes)

Results: 2101
