# Supplementary figures and images for: Adjustment in third culture kids: A systematic review of literature
Source: Front Psychol. 2022 Nov 28;13:939044. doi: 10.3389/fpsyg.2022.939044 (PMC9743971; doi:10.3389/fpsyg.2022.939044)

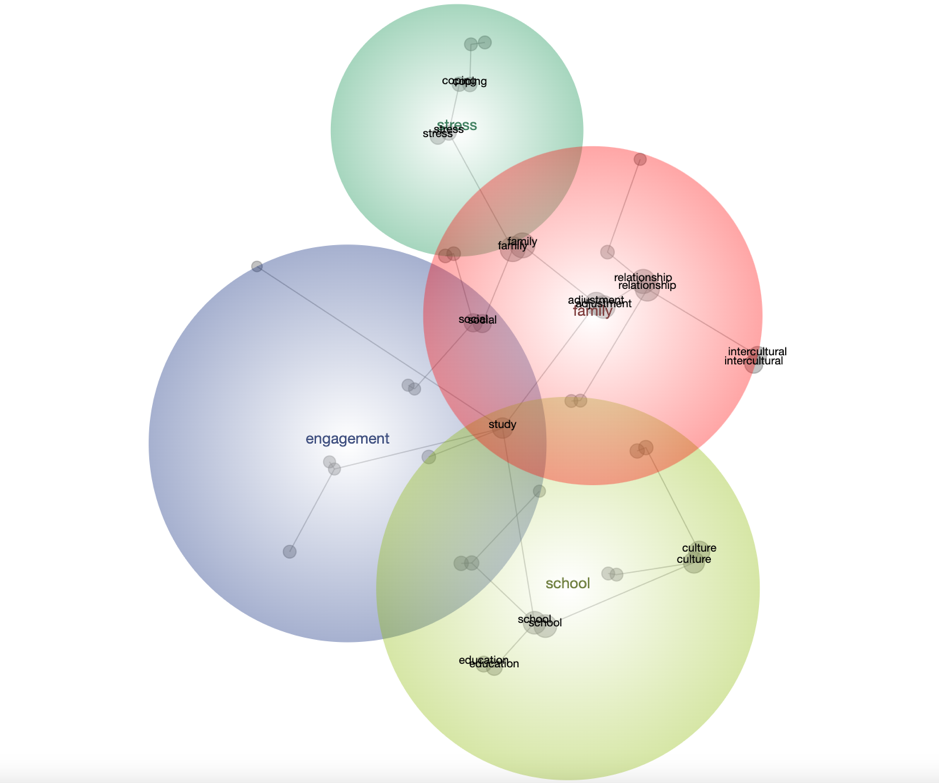

Supplement: Supplementary file 3 [file Image_1.PNG]
